# (6R*,10R*)-Dimethyl 1,4-dioxaspiro­[4.5]decane-6,10-dicarboxyl­ate

**DOI:** 10.1107/S160053681300161X

**Published:** 2013-01-19

**Authors:** Amita Jahangiri, Ola F. Wendt, Daniel Strand

**Affiliations:** a Centre for Analysis and Synthesis, Department of Chemistry, Lund University, Box 124, 221 00 Lund, Sweden; b Centre for Analysis and Synthesis, Department of Chemistry, Lund University, Box 123, 221 00 Lund, Sweden

## Abstract

The title compound, C_12_H_18_O_6_, is in the usual chair conformation with the two ester functions in a 1,3-*trans* orientation. With a value of 1.439 (2) Å, the pseudo-axial C—O bond of the 1,3-dioxolane ring is slightly longer than the corresponding equatorial C—O bond of 1.424 (3) Å. The O—C—O angle of the dioxolane ring is 106.25 (17)°.

## Related literature

The starting material (1*R*,3*S*)-dimethyl 2-oxocyclo­hexane-1,3-dicarboxyl­ate was prepared following a known procedure (Blicke & McCarty, 1959 ▶). Alternative methods for the synthesis of this coumpound include alkyl­ation of cyclo­hexa­none (Balasubrahmanyam & Balasubramanian, 1969 ▶; Beckman & Munshi, 2011 ▶). Synthesis and characterization of a related 1,3-*trans*-dicarboxyl­ate cyclo­hexa­none has been reported (Scaric & Turjak-Cebic, 1982 ▶). The acetal formation follows standard procedures (Wuts & Greene, 2007 ▶).
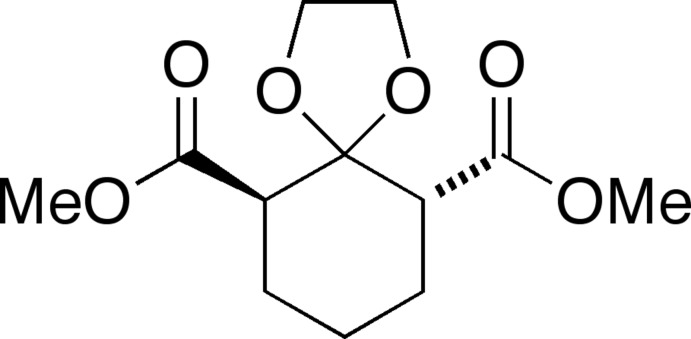


## Experimental

### 

#### Crystal data

C_12_H_18_O_6_
*M* *_r_* = 258.26
Monoclinic, 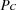

*a* = 8.6243 (9) Å
*b* = 7.3203 (6) Å
*c* = 10.1704 (9) Å
β = 91.719 (8)°
*V* = 641.79 (10) Å^3^
*Z* = 2
Mo *K*α radiation
μ = 0.11 mm^−1^
*T* = 293 K
0.2 × 0.2 × 0.05 mm

#### Data collection

Agilent Xcalibur Sapphire3 diffractometer
Absorption correction: multi-scan (*CrysAlis PRO*; Agilent, 2011 ▶) *T*_min_ = 0.919, *T*_max_ = 1.000
5645 measured reflections
2717 independent reflections
2329 reflections with *I* > 2σ(*I*)
*R*_int_ = 0.025

#### Refinement

*R*[*F* ^2^ > 2σ(*F* ^2^)] = 0.047
*wR*(*F* ^2^) = 0.148
*S* = 1.03
2717 reflections
163 parameters
2 restraints
H-atom parameters constrained
Δρ_max_ = 0.31 e Å^−3^
Δρ_min_ = −0.23 e Å^−3^

### 

Data collection: *CrysAlis PRO* (Agilent, 2011 ▶); cell refinement: *CrysAlis PRO*; data reduction: *CrysAlis PRO*; program(s) used to solve structure: *SHELXS97* (Sheldrick, 2008 ▶); program(s) used to refine structure: *SHELXL97* (Sheldrick, 2008 ▶); molecular graphics: *CrystalMaker* (CrystalMaker, 2011 ▶); software used to prepare material for publication: *SHELXL97*.

## Supplementary Material

Crystal structure: contains datablock(s) I, global. DOI: 10.1107/S160053681300161X/ds2225sup1.cif

Structure factors: contains datablock(s) I. DOI: 10.1107/S160053681300161X/ds2225Isup2.hkl

Supplementary material file. DOI: 10.1107/S160053681300161X/ds2225Isup3.cml

Additional supplementary materials:  crystallographic information; 3D view; checkCIF report

## Figures and Tables

**Fig. 1.**
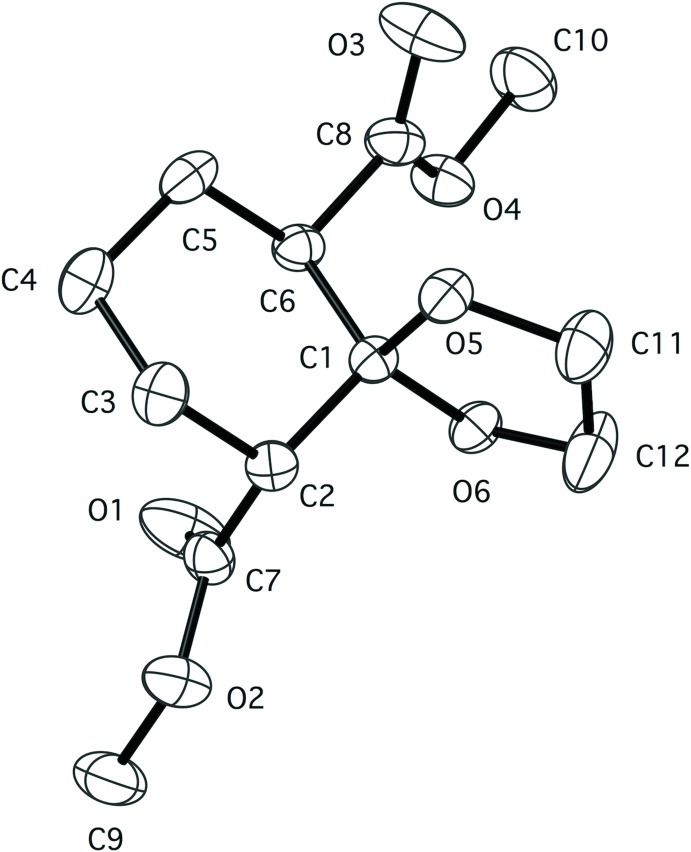
The molecular structure of the title compound with atom labels and 30% probability displacement ellipsoids. H-atoms were omitted for clarity.

## supplementary crystallographic information

### Comment

The cyclohexane ring is in the usual chair conformation. All intramolecular distances and angles display expected values. The dioxolane occupies a pseudo-twist form oriented towards the axial ester group of the cyclohexane ring. Presumably to reduce unfavorable interactions with the carbonyl group of the equatorial ester moiety.

### Experimental

(1*R*,3*S*)-dimethyl 2-oxocyclohexane-1,3-dicarboxylate (0.5 g, 2.5 mmol) was dissolved in toluene (10 mL). Ethylene glycol (1.6 g, 25.7 mmol) and a catalytic amount of *p*-toluene sulfonic acid were then added sequentially. The vessel was fitted with a Dean-Stark trap, heated to reflux for 3 h, and then cooled to RT. The reaction mixture was washed with NaHCO_3_ (10 ml, sat. aq.) and water (10 ml). The organic phase was dried (MgSO_4_), filtered, and concentrated under reduced pressure. ^1^H NMR of the crude shows a single diastereomer. The crude product was purified by flash chromatography (6.25% EtOAc/pet. ether) to give (6*R**,10*R**)dimethyl-1,4-dioxosparo[4,5]decane-6,10-dicarboxylate as a colorless oil (0.30 g, 46%), which crystallized under vaccuum upon standing.

R*_f_*: 0.3 in 6.25% EtOAc/pet. ether ^1^H-NMR: (400 MHz, CDCl_3_) δ: 4.0–3.8 (m, 4H), 3.69 (s, 6H), 3.17 (t, 2H), 2.6 (dd, *J* = 8, 2H), 2.0–1.8 (m, 2H) p.p.m.. ^13^C-NMR: (101 MHz, CDCl_3_) δ: 66.2, 65.1, 52.1, 51.8, 51.7, 47.5, 27.1, 26.6, 23.5, 19.8 p.p.m.. IR: (CHCl_3_, film): 1727 (*s*), 1434 (*m*), 1161 (*s*) cm^-1^.

### Refinement

The H atoms were positioned geometrically and treated as riding on their parent atoms with C–H distances of 0.93–0.97 Å, and *U*_iso_(H) = 1.2 *U*_eq_. The highest difference peak in the Fourier map is located 0.87 Å from C12 and the lowest is located 0.30 Å from H12B.

### Crystal data

**Table:**

C_12_H_18_O_6_	*F*(000) = 276
*M**_r_* = 258.26	*D*_x_ = 1.336 Mg m^−^^3^
Monoclinic, *P**c*	Mo *K*α radiation, λ = 0.71073 Å
Hall symbol: P -2yc	Cell parameters from 1887 reflections
*a* = 8.6243 (9) Å	θ = 2.8–28.6°
*b* = 7.3203 (6) Å	µ = 0.11 mm^−^^1^
*c* = 10.1704 (9) Å	*T* = 293 K
β = 91.719 (8)°	Plate, colourless
*V* = 641.79 (10) Å^3^	0.2 × 0.2 × 0.05 mm
*Z* = 2	

### Data collection

**Table:**

Agilent Xcalibur Sapphire3 diffractometer	2717 independent reflections
Radiation source: Enhance (Mo) X-ray Source	2329 reflections with *I* > 2σ(*I*)
Graphite monochromator	*R*_int_ = 0.025
Detector resolution: 16.1829 pixels mm^-1^	θ_max_ = 28.7°, θ_min_ = 2.8°
ω scans	*h* = −10→10
Absorption correction: multi-scan (*CrysAlis PRO*; Agilent, 2011)	*k* = −9→9
*T*_min_ = 0.919, *T*_max_ = 1.000	*l* = −13→13
5645 measured reflections	

### Refinement

**Table:**

Refinement on *F*^2^	Primary atom site location: structure-invariant direct methods
Least-squares matrix: full	Secondary atom site location: difference Fourier map
*R*[*F*^2^ > 2σ(*F*^2^)] = 0.047	Hydrogen site location: inferred from neighbouring sites
*wR*(*F*^2^) = 0.148	H-atom parameters constrained
*S* = 1.03	*w* = 1/[σ^2^(*F*_o_^2^) + (0.1*P*)^2^] where *P* = (*F*_o_^2^ + 2*F*_c_^2^)/3
2717 reflections	(Δ/σ)_max_ < 0.001
163 parameters	Δρ_max_ = 0.31 e Å^−^^3^
2 restraints	Δρ_min_ = −0.23 e Å^−^^3^

### Special details

**Table:**

Geometry. All e.s.d.'s (except the e.s.d. in the dihedral angle between two l.s. planes) are estimated using the full covariance matrix. The cell e.s.d.'s are taken into account individually in the estimation of e.s.d.'s in distances, angles and torsion angles; correlations between e.s.d.'s in cell parameters are only used when they are defined by crystal symmetry. An approximate (isotropic) treatment of cell e.s.d.'s is used for estimating e.s.d.'s involving l.s. planes.
Refinement. Refinement of *F*^2^ against ALL reflections. The weighted *R*-factor *wR* and goodness of fit *S* are based on *F*^2^, conventional *R*-factors *R* are based on *F*, with *F* set to zero for negative *F*^2^. The threshold expression of *F*^2^ > σ(*F*^2^) is used only for calculating *R*-factors(gt) *etc*. and is not relevant to the choice of reflections for refinement. *R*-factors based on *F*^2^ are statistically about twice as large as those based on *F*, and *R*- factors based on ALL data will be even larger.

### Fractional atomic coordinates and isotropic or equivalent isotropic displacement parameters (Å^2^)

**Table:**

	*x*	*y*	*z*	*U*_iso_*/*U*_eq_	
C1	0.4569 (2)	0.2366 (3)	0.86367 (19)	0.0350 (5)	
O1	0.2305 (3)	0.1361 (4)	1.0635 (2)	0.0837 (8)	
C2	0.2830 (3)	0.1979 (3)	0.8327 (2)	0.0397 (5)	
H2	0.2775	0.1093	0.7605	0.048*	
O2	0.0836 (3)	0.0069 (3)	0.9057 (2)	0.0618 (6)	
C3	0.1999 (3)	0.3712 (4)	0.7846 (3)	0.0527 (6)	
H3A	0.0899	0.3460	0.7733	0.063*	
H3B	0.2388	0.4053	0.6995	0.063*	
O3	0.7232 (3)	0.5536 (4)	0.9408 (3)	0.0847 (8)	
O4	0.7037 (2)	0.3362 (3)	1.09499 (18)	0.0544 (5)	
C4	0.2227 (4)	0.5314 (4)	0.8801 (3)	0.0589 (7)	
H4A	0.1742	0.6400	0.8427	0.071*	
H4B	0.1728	0.5039	0.9620	0.071*	
O5	0.52831 (19)	0.2833 (2)	0.74210 (15)	0.0452 (4)	
C5	0.3944 (4)	0.5677 (3)	0.9073 (3)	0.0535 (7)	
H5A	0.4059	0.6666	0.9703	0.064*	
H5B	0.4420	0.6057	0.8265	0.064*	
O6	0.53266 (19)	0.0753 (2)	0.91042 (16)	0.0431 (4)	
C6	0.4790 (3)	0.3956 (3)	0.9621 (2)	0.0398 (5)	
H6	0.4301	0.3607	1.0441	0.048*	
C7	0.2023 (3)	0.1132 (4)	0.9483 (2)	0.0442 (5)	
C8	0.6476 (3)	0.4376 (4)	0.9939 (2)	0.0473 (6)	
C9	−0.0086 (5)	−0.0765 (5)	1.0061 (4)	0.0781 (10)	
H9A	−0.0899	−0.1481	0.9651	0.117*	
H9B	−0.0533	0.0171	1.0591	0.117*	
H9C	0.0562	−0.1539	1.0606	0.117*	
C10	0.8590 (4)	0.3810 (5)	1.1422 (4)	0.0701 (9)	
H10A	0.8881	0.3015	1.2138	0.105*	
H10B	0.8617	0.5054	1.1719	0.105*	
H10C	0.9301	0.3657	1.0722	0.105*	
C11	0.6241 (5)	0.1332 (5)	0.7054 (3)	0.0690 (8)	
H11A	0.5974	0.0938	0.6165	0.083*	
H11B	0.7326	0.1685	0.7093	0.083*	
C12	0.5965 (6)	−0.0107 (5)	0.7970 (4)	0.0818 (12)	
H12A	0.6926	−0.0730	0.8208	0.098*	
H12B	0.5241	−0.0990	0.7594	0.098*	

### Atomic displacement parameters (Å^2^)

**Table:**

	*U*^11^	*U*^22^	*U*^33^	*U*^12^	*U*^13^	*U*^23^
C1	0.0413 (12)	0.0341 (10)	0.0298 (10)	0.0001 (8)	0.0028 (8)	0.0031 (8)
O1	0.0846 (17)	0.124 (2)	0.0432 (11)	−0.0515 (15)	0.0057 (10)	0.0031 (12)
C2	0.0429 (12)	0.0414 (12)	0.0346 (11)	−0.0009 (9)	−0.0037 (8)	−0.0019 (9)
O2	0.0594 (12)	0.0623 (12)	0.0638 (12)	−0.0207 (9)	0.0007 (9)	−0.0022 (9)
C3	0.0543 (15)	0.0568 (15)	0.0465 (14)	0.0105 (12)	−0.0066 (11)	0.0056 (11)
O3	0.0750 (16)	0.0944 (18)	0.0845 (17)	−0.0420 (15)	0.0001 (12)	0.0270 (14)
O4	0.0454 (10)	0.0592 (11)	0.0580 (11)	−0.0120 (8)	−0.0054 (8)	0.0028 (9)
C4	0.0665 (19)	0.0498 (14)	0.0601 (16)	0.0181 (13)	−0.0012 (13)	0.0003 (13)
O5	0.0594 (11)	0.0409 (8)	0.0360 (8)	0.0035 (7)	0.0103 (7)	0.0046 (7)
C5	0.0700 (19)	0.0319 (11)	0.0586 (16)	0.0019 (11)	0.0038 (13)	−0.0062 (11)
O6	0.0502 (10)	0.0344 (8)	0.0445 (9)	0.0041 (7)	−0.0009 (7)	0.0060 (7)
C6	0.0458 (13)	0.0375 (11)	0.0364 (11)	−0.0049 (9)	0.0048 (9)	−0.0009 (9)
C7	0.0359 (12)	0.0505 (13)	0.0459 (13)	−0.0015 (9)	−0.0030 (9)	−0.0030 (10)
C8	0.0532 (15)	0.0479 (13)	0.0412 (12)	−0.0141 (11)	0.0058 (10)	−0.0059 (10)
C9	0.073 (2)	0.069 (2)	0.093 (3)	−0.0289 (17)	0.0185 (18)	−0.0040 (18)
C10	0.0461 (17)	0.086 (2)	0.078 (2)	−0.0110 (15)	−0.0106 (14)	−0.0074 (18)
C11	0.087 (2)	0.0631 (18)	0.0581 (17)	0.0159 (16)	0.0190 (15)	−0.0070 (15)
C12	0.107 (3)	0.0574 (18)	0.082 (2)	0.0358 (18)	0.031 (2)	0.0060 (16)

### Geometric parameters (Å, º)

**Table:**

C1—O6	1.424 (3)	O5—C11	1.431 (4)
C1—O5	1.439 (2)	C5—C6	1.551 (4)
C1—C6	1.544 (3)	C5—H5A	0.9700
C1—C2	1.549 (3)	C5—H5B	0.9700
O1—C7	1.201 (3)	O6—C12	1.438 (4)
C2—C7	1.516 (4)	C6—C8	1.511 (4)
C2—C3	1.530 (3)	C6—H6	0.9800
C2—H2	0.9800	C9—H9A	0.9600
O2—C7	1.347 (3)	C9—H9B	0.9600
O2—C9	1.448 (4)	C9—H9C	0.9600
C3—C4	1.531 (4)	C10—H10A	0.9600
C3—H3A	0.9700	C10—H10B	0.9600
C3—H3B	0.9700	C10—H10C	0.9600
O3—C8	1.208 (3)	C11—C12	1.431 (5)
O4—C8	1.346 (3)	C11—H11A	0.9700
O4—C10	1.446 (3)	C11—H11B	0.9700
C4—C5	1.522 (4)	C12—H12A	0.9700
C4—H4A	0.9700	C12—H12B	0.9700
C4—H4B	0.9700		
			
O6—C1—O5	106.25 (17)	C8—C6—C5	110.5 (2)
O6—C1—C6	111.21 (17)	C1—C6—C5	109.36 (19)
O5—C1—C6	109.28 (17)	C8—C6—H6	107.9
O6—C1—C2	110.39 (17)	C1—C6—H6	107.9
O5—C1—C2	107.80 (17)	C5—C6—H6	107.9
C6—C1—C2	111.70 (18)	O1—C7—O2	121.6 (2)
C7—C2—C3	111.5 (2)	O1—C7—C2	128.0 (2)
C7—C2—C1	112.39 (17)	O2—C7—C2	110.4 (2)
C3—C2—C1	110.79 (19)	O3—C8—O4	122.8 (2)
C7—C2—H2	107.3	O3—C8—C6	125.2 (3)
C3—C2—H2	107.3	O4—C8—C6	111.9 (2)
C1—C2—H2	107.3	O2—C9—H9A	109.5
C7—O2—C9	116.4 (2)	O2—C9—H9B	109.5
C2—C3—C4	112.48 (19)	H9A—C9—H9B	109.5
C2—C3—H3A	109.1	O2—C9—H9C	109.5
C4—C3—H3A	109.1	H9A—C9—H9C	109.5
C2—C3—H3B	109.1	H9B—C9—H9C	109.5
C4—C3—H3B	109.1	O4—C10—H10A	109.5
H3A—C3—H3B	107.8	O4—C10—H10B	109.5
C8—O4—C10	115.9 (2)	H10A—C10—H10B	109.5
C5—C4—C3	110.8 (2)	O4—C10—H10C	109.5
C5—C4—H4A	109.5	H10A—C10—H10C	109.5
C3—C4—H4A	109.5	H10B—C10—H10C	109.5
C5—C4—H4B	109.5	C12—C11—O5	106.7 (3)
C3—C4—H4B	109.5	C12—C11—H11A	110.4
H4A—C4—H4B	108.1	O5—C11—H11A	110.4
C11—O5—C1	107.9 (2)	C12—C11—H11B	110.4
C4—C5—C6	111.6 (2)	O5—C11—H11B	110.4
C4—C5—H5A	109.3	H11A—C11—H11B	108.6
C6—C5—H5A	109.3	C11—C12—O6	106.0 (3)
C4—C5—H5B	109.3	C11—C12—H12A	110.5
C6—C5—H5B	109.3	O6—C12—H12A	110.5
H5A—C5—H5B	108.0	C11—C12—H12B	110.5
C1—O6—C12	106.2 (2)	O6—C12—H12B	110.5
C8—C6—C1	113.1 (2)	H12A—C12—H12B	108.7
